# 6,12-Bis[(tri­cyclo­hexyl­sil­yl)ethyn­yl]indeno­[1,2-*b*]fluorene

**DOI:** 10.1107/S160053681301218X

**Published:** 2013-05-15

**Authors:** Bradley D. Rose, Lev N. Zakharov, Michael M. Haley

**Affiliations:** a Department of Chemistry and Materials Science Institute, University of Oregon, Eugene, Oregon 97403-1253, USA; b CAMCOR, University of Oregon, 1443 East 13th Avenue, Eugene, Oregon 97403, USA

## Abstract

The title compound, C_60_H_76_Si_2_, a formally anti-aromatic system containing 20-π electrons, contains a rare *p*-xylylene motif. This is displayed by the alternating short and long bonds. The outer rings possess nearly homogenous bond lengths. In the crystal, the molecules forms layers perpendicular to the *c* axis and within these layers there are two one-dimensional stacks with one stack that has a *sp* ^2^ carbon contact of 3.283 (6) Å, less than the sum of the van der Waals radii. The center of the mol­ecule sits on an inversion center.

## Related literature

For the synthetic procedure, see: Kendrick *et al.* (2012 ▶). For information about the indeno­fluorene mol­ecular framework, see: Fix *et al.* (2012 ▶) and about crystal packing, see: Anthony *et al.* (2010 ▶).
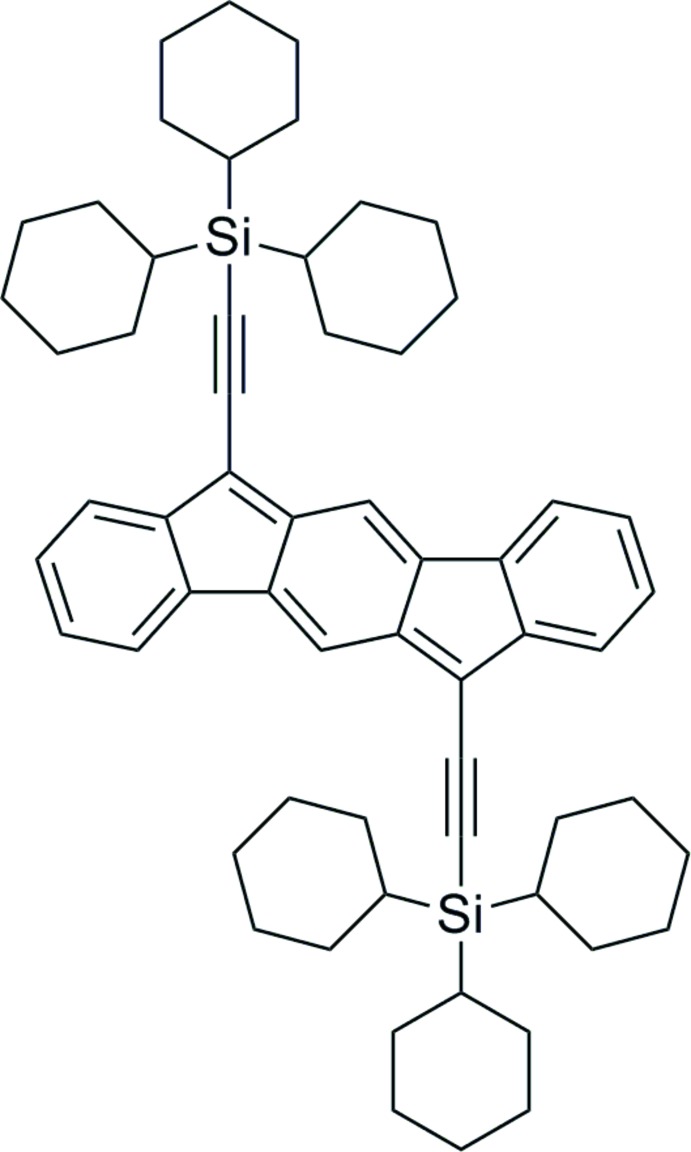


## Experimental

### 

#### Crystal data

C_60_H_76_Si_2_
*M* *_r_* = 853.39
Monoclinic, 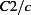

*a* = 20.219 (7) Å
*b* = 7.246 (2) Å
*c* = 33.885 (11) Å
β = 103.017 (7)°
*V* = 4837 (3) Å^3^
*Z* = 4
Mo *K*α radiation
μ = 0.11 mm^−1^
*T* = 193 K
0.08 × 0.03 × 0.01 mm

#### Data collection

Bruker APEX CCD area-detector diffractometer
Absorption correction: multi-scan (*SADABS*; Bruker, 2000 ▶) *T*_min_ = 0.991, *T*_max_ = 0.999
22528 measured reflections
4268 independent reflections
2181 reflections with *I* > 2σ(*I*)
*R*_int_ = 0.176

#### Refinement

*R*[*F* ^2^ > 2σ(*F* ^2^)] = 0.076
*wR*(*F* ^2^) = 0.151
*S* = 1.01
4268 reflections
280 parameters
H-atom parameters constrained
Δρ_max_ = 0.27 e Å^−3^
Δρ_min_ = −0.28 e Å^−3^

### 

Data collection: *SMART* (Bruker, 2000 ▶); cell refinement: *SAINT* (Bruker, 2000 ▶); data reduction: *SAINT*; program(s) used to solve structure: *XS* in *SHELXTL* (Sheldrick, 2008 ▶); program(s) used to refine structure: *XL* in *SHELXTL*; molecular graphics: *XP* in *SHELXTL*; software used to prepare material for publication: *SHELXTL*.

## Supplementary Material

Crystal structure: contains datablock(s) I, global. DOI: 10.1107/S160053681301218X/pk2470sup1.cif

Structure factors: contains datablock(s) I. DOI: 10.1107/S160053681301218X/pk2470Isup2.hkl

Supplementary material file. DOI: 10.1107/S160053681301218X/pk2470Isup3.cml

Additional supplementary materials:  crystallographic information; 3D view; checkCIF report

## Figures and Tables

**Fig. 1.**
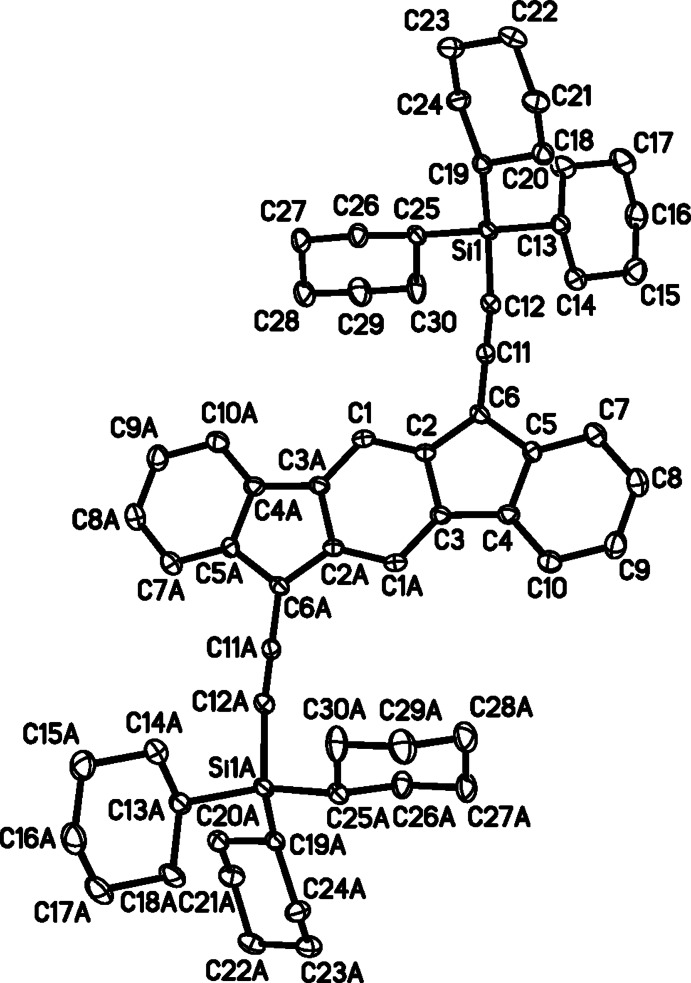
The crystal structure of the title compound. ellipsoids drawn with with 30% probability displacement ellipsoids and the atom-numbering scheme. [Symmetry code (A): 1 - x, -y, 2 - z].

**Fig. 2.**
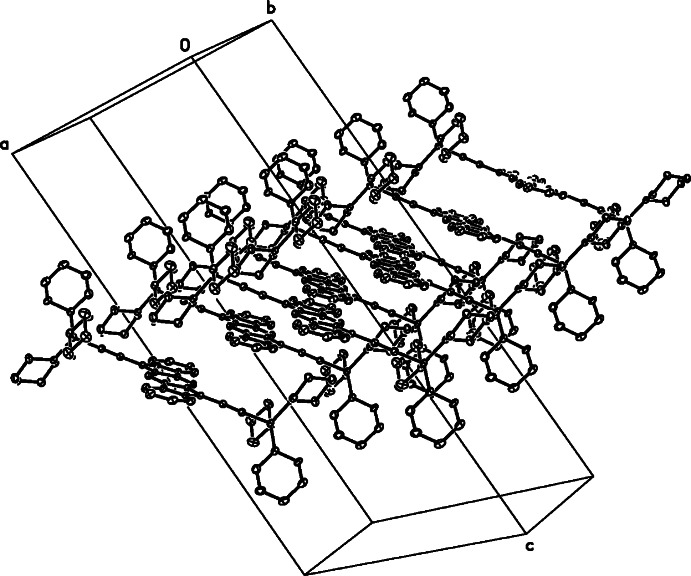
View of molecular stacks of the title compound.

## supplementary crystallographic information

### Comment

Organic electronics are a burgeoning technology that may allow mass printable inexpensive electronic devices such as photovoltaics, organic thin film transistors (OTFT), and light emitting diodes (Anthony, *et al.* 2010). The design of these materials is difficult as there are many factors that affect device performance, for example, the solid state molecular packing and energy levels of the frontier orbitals; thus, the title compound was made to explore this and the solid state structure explored *via* X-ray crystallography. The main findings from this study are that there are no close intermolecular contacts between the *sp*^2^ hybridized carbon atoms. Ideally for a small molecule OFET material, there would be multiple close C—C contacts which are less than the sum of the van der Waals radii (3.4 Å) between the carbon scaffold to allow for efficient charge transport in mulitple dimensions. One of the closest *sp*^2^ C—C intermolecular contact [C7(x,y,z)···C7(1.5-x,-0.5-y,2-z) = 3.283 (6) Å] is found to be sub van der Waals in one dimension. This molecule also provides an example of a crystallographically characterized *p*-xylylene motif that posessed bond length alternation.

### Experimental

Compound was synthesized according to literature procedure (Kendrick, *et al.* 2012). A single-crystal was grown from slow evaporation of chloroform.

### Refinement

H atoms were refined in calculated positions in a rigid group model, C—H = 1.2*U*_eq_(C) for –CH_2_ and –CH groups.

### Crystal data

**Table:**

C_60_H_76_Si_2_	*F*(000) = 1856
*M**_r_* = 853.39	*D*_x_ = 1.172 Mg m^−^^3^
Monoclinic, *C*2/*c*	Mo *K*α radiation, λ = 0.71073 Å
Hall symbol: -C 2yc	Cell parameters from 547 reflections
*a* = 20.219 (7) Å	θ = 2.6–15.4°
*b* = 7.246 (2) Å	µ = 0.11 mm^−^^1^
*c* = 33.885 (11) Å	*T* = 193 K
β = 103.017 (7)°	Block, purple
*V* = 4837 (3) Å^3^	0.08 × 0.03 × 0.01 mm
*Z* = 4	

### Data collection

**Table:**

Bruker APEX CCD area-detector diffractometer	4268 independent reflections
Radiation source: fine-focus sealed tube	2181 reflections with *I* > 2σ(*I*)
Graphite monochromator	*R*_int_ = 0.176
φ and ω scans	θ_max_ = 25.0°, θ_min_ = 2.1°
Absorption correction: multi-scan (*SADABS*; Bruker, 2000)	*h* = −24→24
*T*_min_ = 0.991, *T*_max_ = 0.999	*k* = −8→8
22528 measured reflections	*l* = −40→40

### Refinement

**Table:**

Refinement on *F*^2^	Primary atom site location: structure-invariant direct methods
Least-squares matrix: full	Secondary atom site location: difference Fourier map
*R*[*F*^2^ > 2σ(*F*^2^)] = 0.076	Hydrogen site location: inferred from neighbouring sites
*wR*(*F*^2^) = 0.151	H-atom parameters constrained
*S* = 1.01	*w* = 1/[σ^2^(*F*_o_^2^) + (0.0419*P*)^2^] where *P* = (*F*_o_^2^ + 2*F*_c_^2^)/3
4268 reflections	(Δ/σ)_max_ = 0.001
280 parameters	Δρ_max_ = 0.27 e Å^−^^3^
0 restraints	Δρ_min_ = −0.28 e Å^−^^3^

### Special details

**Table:**

Geometry. All e.s.d.'s (except the e.s.d. in the dihedral angle between two l.s. planes) are estimated using the full covariance matrix. The cell e.s.d.'s are taken into account individually in the estimation of e.s.d.'s in distances, angles and torsion angles; correlations between e.s.d.'s in cell parameters are only used when they are defined by crystal symmetry. An approximate (isotropic) treatment of cell e.s.d.'s is used for estimating e.s.d.'s involving l.s. planes.
Refinement. Refinement of *F*^2^ against ALL reflections. The weighted *R*-factor *wR* and goodness of fit *S* are based on *F*^2^, conventional *R*-factors *R* are based on *F*, with *F* set to zero for negative *F*^2^. The threshold expression of *F*^2^ > σ(*F*^2^) is used only for calculating *R*-factors(gt) *etc*. and is not relevant to the choice of reflections for refinement. *R*-factors based on *F*^2^ are statistically about twice as large as those based on *F*, and *R*- factors based on ALL data will be even larger.

### Fractional atomic coordinates and isotropic or equivalent isotropic displacement parameters (Å^2^)

**Table:**

	*x*	*y*	*z*	*U*_iso_*/*U*_eq_	
Si1	0.64679 (6)	0.27756 (16)	0.85715 (3)	0.0270 (3)	
C1	0.5070 (2)	0.1499 (5)	0.97336 (11)	0.0288 (10)	
H1A	0.5123	0.2479	0.9557	0.035*	
C2	0.5493 (2)	−0.0092 (5)	0.97752 (11)	0.0246 (10)	
C3	0.5413 (2)	−0.1567 (5)	1.00438 (11)	0.0260 (10)	
C4	0.5924 (2)	−0.2973 (6)	1.00215 (11)	0.0286 (10)	
C5	0.63048 (19)	−0.2320 (5)	0.97507 (10)	0.0244 (9)	
C6	0.60219 (19)	−0.0535 (5)	0.95896 (11)	0.0249 (10)	
C7	0.6839 (2)	−0.3316 (6)	0.96734 (12)	0.0347 (11)	
H7A	0.7103	−0.2852	0.9496	0.042*	
C8	0.6983 (2)	−0.5026 (6)	0.98614 (13)	0.0391 (12)	
H8A	0.7350	−0.5739	0.9812	0.047*	
C9	0.6603 (2)	−0.5689 (6)	1.01174 (13)	0.0415 (12)	
H9A	0.6703	−0.6870	1.0238	0.050*	
C10	0.6070 (2)	−0.4658 (6)	1.02035 (12)	0.0362 (11)	
H10A	0.5813	−0.5116	1.0385	0.043*	
C11	0.6207 (2)	0.0464 (5)	0.92737 (12)	0.0270 (10)	
C12	0.63409 (19)	0.1298 (6)	0.89918 (12)	0.0280 (10)	
C13	0.6609 (2)	0.1232 (5)	0.81513 (11)	0.0298 (10)	
H13A	0.7043	0.0559	0.8263	0.036*	
C14	0.6076 (2)	−0.0260 (6)	0.80074 (13)	0.0441 (13)	
H14A	0.6024	−0.1011	0.8243	0.053*	
H14B	0.5634	0.0337	0.7893	0.053*	
C15	0.6257 (3)	−0.1525 (6)	0.76890 (13)	0.0506 (14)	
H15A	0.6669	−0.2239	0.7812	0.061*	
H15B	0.5882	−0.2411	0.7595	0.061*	
C16	0.6381 (2)	−0.0457 (6)	0.73328 (13)	0.0490 (13)	
H16A	0.5952	0.0117	0.7186	0.059*	
H16B	0.6539	−0.1307	0.7144	0.059*	
C17	0.6908 (3)	0.1026 (7)	0.74695 (13)	0.0551 (14)	
H17A	0.6956	0.1765	0.7232	0.066*	
H17B	0.7351	0.0436	0.7584	0.066*	
C18	0.6726 (2)	0.2297 (6)	0.77841 (12)	0.0451 (13)	
H18A	0.6310	0.2994	0.7660	0.054*	
H18B	0.7098	0.3198	0.7874	0.054*	
C19	0.72462 (19)	0.4184 (5)	0.87874 (12)	0.0272 (10)	
H19A	0.7183	0.4628	0.9056	0.033*	
C20	0.7901 (2)	0.3061 (5)	0.88827 (12)	0.0336 (11)	
H20A	0.7841	0.1995	0.9054	0.040*	
H20B	0.7990	0.2574	0.8627	0.040*	
C21	0.8510 (2)	0.4179 (6)	0.90996 (13)	0.0396 (12)	
H21A	0.8924	0.3405	0.9141	0.048*	
H21B	0.8446	0.4559	0.9369	0.048*	
C22	0.8603 (2)	0.5878 (6)	0.88565 (13)	0.0415 (12)	
H22A	0.8988	0.6617	0.9010	0.050*	
H22B	0.8714	0.5494	0.8599	0.050*	
C23	0.7967 (2)	0.7052 (6)	0.87667 (13)	0.0435 (12)	
H23A	0.8033	0.8104	0.8593	0.052*	
H23B	0.7887	0.7557	0.9023	0.052*	
C24	0.7351 (2)	0.5942 (5)	0.85547 (13)	0.0372 (12)	
H24A	0.7405	0.5593	0.8281	0.045*	
H24B	0.6941	0.6726	0.8521	0.045*	
C25	0.5693 (2)	0.4297 (6)	0.84335 (12)	0.0308 (11)	
H25A	0.5749	0.5091	0.8202	0.037*	
C26	0.5644 (2)	0.5583 (6)	0.87825 (13)	0.0390 (12)	
H26A	0.6055	0.6367	0.8847	0.047*	
H26B	0.5634	0.4832	0.9025	0.047*	
C27	0.5016 (2)	0.6826 (6)	0.86891 (15)	0.0476 (13)	
H27A	0.4994	0.7555	0.8933	0.057*	
H27B	0.5053	0.7699	0.8470	0.057*	
C28	0.4383 (2)	0.5714 (6)	0.85617 (15)	0.0498 (13)	
H28A	0.3988	0.6555	0.8489	0.060*	
H28B	0.4321	0.4938	0.8791	0.060*	
C29	0.4407 (2)	0.4491 (7)	0.82035 (15)	0.0549 (14)	
H29A	0.4420	0.5270	0.7965	0.066*	
H29B	0.3992	0.3725	0.8137	0.066*	
C30	0.5032 (2)	0.3236 (6)	0.82947 (14)	0.0494 (14)	
H30A	0.4986	0.2343	0.8508	0.059*	
H30B	0.5051	0.2526	0.8048	0.059*	

### Atomic displacement parameters (Å^2^)

**Table:**

	*U*^11^	*U*^22^	*U*^33^	*U*^12^	*U*^13^	*U*^23^
Si1	0.0259 (7)	0.0320 (7)	0.0262 (6)	0.0027 (6)	0.0122 (5)	0.0036 (6)
C1	0.032 (3)	0.028 (3)	0.027 (2)	−0.006 (2)	0.007 (2)	0.0055 (19)
C2	0.026 (2)	0.026 (2)	0.022 (2)	−0.001 (2)	0.0062 (19)	0.0042 (19)
C3	0.028 (2)	0.028 (2)	0.023 (2)	−0.003 (2)	0.009 (2)	0.0007 (19)
C4	0.034 (3)	0.029 (3)	0.025 (2)	−0.002 (2)	0.009 (2)	0.003 (2)
C5	0.026 (2)	0.027 (2)	0.020 (2)	0.000 (2)	0.0050 (18)	−0.0040 (19)
C6	0.024 (2)	0.030 (3)	0.022 (2)	−0.003 (2)	0.009 (2)	−0.003 (2)
C7	0.035 (3)	0.043 (3)	0.029 (3)	0.002 (2)	0.011 (2)	−0.001 (2)
C8	0.040 (3)	0.045 (3)	0.033 (3)	0.009 (2)	0.010 (2)	−0.004 (2)
C9	0.050 (3)	0.037 (3)	0.038 (3)	0.013 (2)	0.011 (3)	0.004 (2)
C10	0.042 (3)	0.038 (3)	0.031 (3)	0.001 (2)	0.014 (2)	0.005 (2)
C11	0.026 (2)	0.027 (3)	0.029 (2)	0.002 (2)	0.009 (2)	−0.002 (2)
C12	0.024 (2)	0.032 (3)	0.028 (3)	0.001 (2)	0.007 (2)	0.000 (2)
C13	0.029 (3)	0.033 (3)	0.028 (2)	0.005 (2)	0.010 (2)	0.004 (2)
C14	0.051 (3)	0.041 (3)	0.047 (3)	0.000 (2)	0.026 (3)	−0.005 (2)
C15	0.071 (4)	0.043 (3)	0.040 (3)	0.002 (3)	0.016 (3)	−0.009 (2)
C16	0.061 (3)	0.053 (3)	0.035 (3)	0.010 (3)	0.014 (3)	−0.007 (3)
C17	0.067 (4)	0.068 (4)	0.039 (3)	−0.001 (3)	0.032 (3)	−0.005 (3)
C18	0.063 (3)	0.052 (3)	0.027 (2)	−0.009 (3)	0.025 (2)	−0.006 (2)
C19	0.029 (2)	0.031 (3)	0.024 (2)	0.002 (2)	0.011 (2)	0.001 (2)
C20	0.037 (3)	0.036 (3)	0.031 (3)	0.003 (2)	0.012 (2)	0.002 (2)
C21	0.030 (3)	0.047 (3)	0.041 (3)	−0.006 (2)	0.005 (2)	0.002 (2)
C22	0.029 (3)	0.056 (3)	0.042 (3)	−0.008 (2)	0.012 (2)	0.004 (2)
C23	0.042 (3)	0.042 (3)	0.049 (3)	−0.009 (3)	0.016 (2)	0.002 (3)
C24	0.032 (3)	0.033 (3)	0.046 (3)	−0.001 (2)	0.009 (2)	0.009 (2)
C25	0.029 (3)	0.033 (3)	0.033 (3)	0.002 (2)	0.014 (2)	0.001 (2)
C26	0.026 (3)	0.035 (3)	0.055 (3)	0.001 (2)	0.008 (2)	−0.011 (2)
C27	0.029 (3)	0.040 (3)	0.078 (4)	0.005 (2)	0.020 (3)	−0.009 (3)
C28	0.032 (3)	0.051 (3)	0.069 (4)	0.010 (2)	0.016 (3)	−0.006 (3)
C29	0.033 (3)	0.064 (4)	0.065 (4)	0.005 (3)	0.006 (3)	−0.013 (3)
C30	0.031 (3)	0.053 (3)	0.059 (3)	0.014 (2)	−0.001 (2)	−0.019 (3)

### Geometric parameters (Å, º)

**Table:**

Si1—C12	1.845 (4)	C17—H17B	0.9900
Si1—C19	1.880 (4)	C18—H18A	0.9900
Si1—C13	1.882 (4)	C18—H18B	0.9900
Si1—C25	1.887 (4)	C19—C20	1.525 (5)
C1—C3^i^	1.364 (5)	C19—C24	1.537 (5)
C1—C2	1.423 (5)	C19—H19A	1.0000
C1—H1A	0.9500	C20—C21	1.519 (5)
C2—C6	1.395 (5)	C20—H20A	0.9900
C2—C3	1.437 (5)	C20—H20B	0.9900
C3—C1^i^	1.364 (5)	C21—C22	1.516 (5)
C3—C4	1.465 (5)	C21—H21A	0.9900
C4—C10	1.370 (5)	C21—H21B	0.9900
C4—C5	1.406 (5)	C22—C23	1.514 (5)
C5—C7	1.372 (5)	C22—H22A	0.9900
C5—C6	1.469 (5)	C22—H22B	0.9900
C6—C11	1.411 (5)	C23—C24	1.521 (5)
C7—C8	1.394 (5)	C23—H23A	0.9900
C7—H7A	0.9500	C23—H23B	0.9900
C8—C9	1.370 (5)	C24—H24A	0.9900
C8—H8A	0.9500	C24—H24B	0.9900
C9—C10	1.394 (5)	C25—C30	1.521 (5)
C9—H9A	0.9500	C25—C26	1.527 (5)
C10—H10A	0.9500	C25—H25A	1.0000
C11—C12	1.211 (5)	C26—C27	1.530 (5)
C13—C14	1.528 (5)	C26—H26A	0.9900
C13—C18	1.528 (5)	C26—H26B	0.9900
C13—H13A	1.0000	C27—C28	1.492 (6)
C14—C15	1.522 (5)	C27—H27A	0.9900
C14—H14A	0.9900	C27—H27B	0.9900
C14—H14B	0.9900	C28—C29	1.512 (6)
C15—C16	1.502 (6)	C28—H28A	0.9900
C15—H15A	0.9900	C28—H28B	0.9900
C15—H15B	0.9900	C29—C30	1.531 (5)
C16—C17	1.510 (6)	C29—H29A	0.9900
C16—H16A	0.9900	C29—H29B	0.9900
C16—H16B	0.9900	C30—H30A	0.9900
C17—C18	1.515 (5)	C30—H30B	0.9900
C17—H17A	0.9900		
			
C12—Si1—C19	105.33 (18)	H18A—C18—H18B	107.9
C12—Si1—C13	108.07 (18)	C20—C19—C24	109.7 (3)
C19—Si1—C13	111.14 (18)	C20—C19—Si1	113.8 (3)
C12—Si1—C25	106.13 (18)	C24—C19—Si1	116.7 (3)
C19—Si1—C25	110.79 (18)	C20—C19—H19A	105.1
C13—Si1—C25	114.77 (19)	C24—C19—H19A	105.1
C3^i^—C1—C2	117.7 (4)	Si1—C19—H19A	105.1
C3^i^—C1—H1A	121.2	C21—C20—C19	112.9 (3)
C2—C1—H1A	121.2	C21—C20—H20A	109.0
C6—C2—C1	130.3 (4)	C19—C20—H20A	109.0
C6—C2—C3	108.7 (3)	C21—C20—H20B	109.0
C1—C2—C3	121.0 (3)	C19—C20—H20B	109.0
C1^i^—C3—C2	121.3 (4)	H20A—C20—H20B	107.8
C1^i^—C3—C4	131.0 (4)	C20—C21—C22	110.9 (3)
C2—C3—C4	107.7 (3)	C20—C21—H21A	109.5
C10—C4—C5	120.0 (4)	C22—C21—H21A	109.5
C10—C4—C3	132.8 (4)	C20—C21—H21B	109.5
C5—C4—C3	107.2 (3)	C22—C21—H21B	109.5
C7—C5—C4	121.1 (4)	H21A—C21—H21B	108.1
C7—C5—C6	130.5 (4)	C23—C22—C21	111.2 (3)
C4—C5—C6	108.3 (3)	C23—C22—H22A	109.4
C2—C6—C11	125.8 (4)	C21—C22—H22A	109.4
C2—C6—C5	108.0 (3)	C23—C22—H22B	109.4
C11—C6—C5	125.9 (3)	C21—C22—H22B	109.4
C5—C7—C8	118.3 (4)	H22A—C22—H22B	108.0
C5—C7—H7A	120.9	C22—C23—C24	111.5 (4)
C8—C7—H7A	120.9	C22—C23—H23A	109.3
C9—C8—C7	120.8 (4)	C24—C23—H23A	109.3
C9—C8—H8A	119.6	C22—C23—H23B	109.3
C7—C8—H8A	119.6	C24—C23—H23B	109.3
C8—C9—C10	121.0 (4)	H23A—C23—H23B	108.0
C8—C9—H9A	119.5	C23—C24—C19	112.9 (3)
C10—C9—H9A	119.5	C23—C24—H24A	109.0
C4—C10—C9	118.8 (4)	C19—C24—H24A	109.0
C4—C10—H10A	120.6	C23—C24—H24B	109.0
C9—C10—H10A	120.6	C19—C24—H24B	109.0
C12—C11—C6	177.3 (4)	H24A—C24—H24B	107.8
C11—C12—Si1	173.1 (4)	C30—C25—C26	110.1 (3)
C14—C13—C18	109.0 (3)	C30—C25—Si1	113.8 (3)
C14—C13—Si1	116.4 (3)	C26—C25—Si1	111.1 (3)
C18—C13—Si1	113.2 (3)	C30—C25—H25A	107.2
C14—C13—H13A	105.8	C26—C25—H25A	107.2
C18—C13—H13A	105.8	Si1—C25—H25A	107.2
Si1—C13—H13A	105.8	C25—C26—C27	113.2 (4)
C15—C14—C13	112.8 (4)	C25—C26—H26A	108.9
C15—C14—H14A	109.0	C27—C26—H26A	108.9
C13—C14—H14A	109.0	C25—C26—H26B	108.9
C15—C14—H14B	109.0	C27—C26—H26B	108.9
C13—C14—H14B	109.0	H26A—C26—H26B	107.7
H14A—C14—H14B	107.8	C28—C27—C26	111.1 (4)
C16—C15—C14	111.7 (4)	C28—C27—H27A	109.4
C16—C15—H15A	109.3	C26—C27—H27A	109.4
C14—C15—H15A	109.3	C28—C27—H27B	109.4
C16—C15—H15B	109.3	C26—C27—H27B	109.4
C14—C15—H15B	109.3	H27A—C27—H27B	108.0
H15A—C15—H15B	107.9	C27—C28—C29	111.6 (4)
C15—C16—C17	110.7 (4)	C27—C28—H28A	109.3
C15—C16—H16A	109.5	C29—C28—H28A	109.3
C17—C16—H16A	109.5	C27—C28—H28B	109.3
C15—C16—H16B	109.5	C29—C28—H28B	109.3
C17—C16—H16B	109.5	H28A—C28—H28B	108.0
H16A—C16—H16B	108.1	C28—C29—C30	111.2 (4)
C16—C17—C18	112.5 (4)	C28—C29—H29A	109.4
C16—C17—H17A	109.1	C30—C29—H29A	109.4
C18—C17—H17A	109.1	C28—C29—H29B	109.4
C16—C17—H17B	109.1	C30—C29—H29B	109.4
C18—C17—H17B	109.1	H29A—C29—H29B	108.0
H17A—C17—H17B	107.8	C25—C30—C29	112.9 (4)
C17—C18—C13	111.8 (4)	C25—C30—H30A	109.0
C17—C18—H18A	109.2	C29—C30—H30A	109.0
C13—C18—H18A	109.2	C25—C30—H30B	109.0
C17—C18—H18B	109.2	C29—C30—H30B	109.0
C13—C18—H18B	109.2	H30A—C30—H30B	107.8
			
C3^i^—C1—C2—C6	−179.9 (4)	C19—Si1—C13—C18	−64.5 (4)
C3^i^—C1—C2—C3	−0.3 (6)	C25—Si1—C13—C18	62.2 (4)
C6—C2—C3—C1^i^	180.0 (4)	C18—C13—C14—C15	54.6 (5)
C1—C2—C3—C1^i^	0.3 (6)	Si1—C13—C14—C15	−175.9 (3)
C6—C2—C3—C4	0.4 (4)	C13—C14—C15—C16	−55.5 (5)
C1—C2—C3—C4	−179.3 (3)	C14—C15—C16—C17	53.9 (5)
C1^i^—C3—C4—C10	2.0 (8)	C15—C16—C17—C18	−54.7 (5)
C2—C3—C4—C10	−178.5 (4)	C16—C17—C18—C13	55.9 (5)
C1^i^—C3—C4—C5	−178.5 (4)	C14—C13—C18—C17	−54.4 (5)
C2—C3—C4—C5	1.1 (4)	Si1—C13—C18—C17	174.3 (3)
C10—C4—C5—C7	−2.1 (6)	C12—Si1—C19—C20	69.1 (3)
C3—C4—C5—C7	178.3 (4)	C13—Si1—C19—C20	−47.7 (3)
C10—C4—C5—C6	177.6 (4)	C25—Si1—C19—C20	−176.5 (3)
C3—C4—C5—C6	−2.0 (4)	C12—Si1—C19—C24	−161.5 (3)
C1—C2—C6—C11	−7.7 (7)	C13—Si1—C19—C24	81.7 (3)
C3—C2—C6—C11	172.6 (4)	C25—Si1—C19—C24	−47.1 (3)
C1—C2—C6—C5	178.1 (4)	C24—C19—C20—C21	53.6 (4)
C3—C2—C6—C5	−1.6 (4)	Si1—C19—C20—C21	−173.6 (3)
C7—C5—C6—C2	−178.0 (4)	C19—C20—C21—C22	−56.2 (5)
C4—C5—C6—C2	2.3 (4)	C20—C21—C22—C23	56.1 (5)
C7—C5—C6—C11	7.8 (7)	C21—C22—C23—C24	−55.3 (5)
C4—C5—C6—C11	−171.9 (4)	C22—C23—C24—C19	54.2 (5)
C4—C5—C7—C8	1.9 (6)	C20—C19—C24—C23	−52.4 (5)
C6—C5—C7—C8	−177.8 (4)	Si1—C19—C24—C23	176.3 (3)
C5—C7—C8—C9	−0.2 (6)	C12—Si1—C25—C30	−60.6 (3)
C7—C8—C9—C10	−1.4 (7)	C19—Si1—C25—C30	−174.4 (3)
C5—C4—C10—C9	0.6 (6)	C13—Si1—C25—C30	58.7 (4)
C3—C4—C10—C9	−179.9 (4)	C12—Si1—C25—C26	64.3 (3)
C8—C9—C10—C4	1.1 (7)	C19—Si1—C25—C26	−49.5 (3)
C2—C6—C11—C12	−69 (9)	C13—Si1—C25—C26	−176.4 (3)
C5—C6—C11—C12	104 (9)	C30—C25—C26—C27	−52.0 (5)
C6—C11—C12—Si1	75 (10)	Si1—C25—C26—C27	−179.0 (3)
C19—Si1—C12—C11	99 (3)	C25—C26—C27—C28	54.7 (5)
C13—Si1—C12—C11	−142 (3)	C26—C27—C28—C29	−55.9 (5)
C25—Si1—C12—C11	−19 (3)	C27—C28—C29—C30	56.0 (6)
C12—Si1—C13—C14	52.9 (4)	C26—C25—C30—C29	51.8 (5)
C19—Si1—C13—C14	168.0 (3)	Si1—C25—C30—C29	177.3 (3)
C25—Si1—C13—C14	−65.3 (4)	C28—C29—C30—C25	−54.4 (5)
C12—Si1—C13—C18	−179.6 (3)		

Symmetry code: (i) −*x*+1, −*y*, −*z*+2.
